# Whiplash-Associated Disorders. Biopsychosocial Profiles of Pain Perception in Forensic Cases of Victims of Motor Vehicle Accidents

**DOI:** 10.3389/fpsyg.2021.716513

**Published:** 2021-08-16

**Authors:** David Pina, Esteban Puente-López, José Antonio Ruiz-Hernández, Aurelio Luna Ruiz-Cabello, Luis Aguerrevere, Teresa Magalhães

**Affiliations:** ^1^Servicio Externo de Ciencias y Técnicas Forenses, Universidad de Murcia, Murcia, Spain; ^2^Facultad de Medicina, Universidad de Murcia, Murcia, Spain; ^3^Department of Human Services, Stephen F. Austin State University, Nacogdoches, TX, United States; ^4^Faculdade de Medicina, Universidade do Porto, Porto, Portugal

**Keywords:** whiplash related injury, biopsychosocial model, multivariate analysis, forensic context, pain research

## Abstract

In order to make a complete diagnosis of all the factors influencing whiplash associated disorders (WAD), the evidence suggests that the condition evaluation should follow an integrated biopsychosocial model. This perspective would offer a fuller view of it, recognizing the interplay between the medical, biomechanical, social, and psychological factors. Despite the progress made in the subject, evidence of which psychosocial factors influence the experience of pain in litigant WAD patients is limited. A cross-sectional design and a cluster analysis was used to study the experience of pain and the psychosocial factors included therein in 249 patients with WAD assessed after suffering a motor vehicle accident. Three clusters were obtained: C1, with low scores of pain and a slight-moderate alteration of the Health-Related Quality of Life (HRQoL); C2, with medium scores of pain, alteration of HRQoL and a perception of moderate disability; and C3, with medium-high scores of pain, alteration of the HQoL, perception of moderate disability, presence of anxious-depressive symptomatology, poorer comprehension of the condition suffered, and the belief that it will extend over a long period of time. The results show a heterogeneous experience of pain in WAD, compatible with the biopsychosocial model of disease and the multidimensional approach to pain. The role of the psychologist in the evaluation of the condition could be useful to obtain a complete view of the condition, thus ensuring that the treatment is adapted to the needs of the patient.

## Introduction

The term whiplash refers to an injury caused by a sudden acceleration/deceleration movement that generates hyperextension in the cervical region. This type of injury causes physical damage, and triggers a wide spectrum of symptoms, collected under the term of Whiplash Associated Disorders (WAD; Spitzer et al., 1995; Pastakia and Kumar, 2011). As Turk et al. (2018) stated, WAD is one of the most common consequences of motor vehicle accidents (MVA), with an estimated prevalence in the population of 200 out of every 100,000 people. The frequency of the condition varies between countries depending on the number of vehicles/inhabitants, road rules, and economic compensation system. Although no up-to-date data are available, it is estimated, for example, that its annual incidence varies between 16 and 200 cases per 100,000 inhabitants, with a high variability depending on the country. For example, in Spain, it amounts to 60.2 cases per 100,000 inhabitants; in Canada, it reaches 70 per 100,000; in Australia, 106 per 100,000; and in the Netherlands, it ranges from 188 to 325 per 100,000 (Pastakia and Kumar, 2011; Regal Ramos, 2011). The economic cost of WAD stands at approximately $42 billion in the United States, and 10 billion euros per year in Europe, with estimates of approximately 3 billion pounds per year in the United Kingdom, and 10 million euros in Spain (Crouch et al., 2006; Kamper et al., 2008; Pink et al., 2016). WAD is also considered to be one of the world’s leading sources of disability (Kamper et al., 2008). Specifically, cervical pain of traumatic and non-traumatic origin is the fourth global cause of disability, with the same range as in 1990, suggesting that recent research on the prevention and rehabilitation of this type of injury has had little effect on its overall impact (Walton and Elliott, 2017).

It is estimated that the high incidence of this condition is because the main etiology of WAD is the cervical hyperextension related to MVA. These cases are frequently associated with a compensation procedure, which promotes the demand for compensation and makes the risk of malingering a potential problem in the forensic and insurance medicine context (Greve et al., 2009). Also, WAD is difficult to evaluate due to the absence of objective injuries evaluable with the available methods and is diagnosed mainly based on the patients’ symptoms. Besides, there are few signs and those that can be found, such as cervical rectifications or muscle contractures, are unspecific because they are common to multiple health conditions with high prevalence (Represas, 2017).

In fact, MVA victims involved in what is known as “compensation-related factors” are believed to overreport symptoms, and a worse illness situation (Rasmussen et al., 2008). This has created a situation of mistrust toward patients who express more serious symptoms than usual, making medico-legal assessment of WAD a challenge, because the line that separates the genuine patient from the malingerer can be blurred (Spearing et al., 2012). However, when evaluating a WAD patient in the context of litigation, it should be taken into account that there are multiple pathways to symptom overreporting, and malingering is just one of them. For example, symptom overreporting may be due to personality traits, symptom misinformation (patients have erroneous information about the condition affecting their experience), presence of a pre-accident condition that aggravates the manifestation of symptoms, or various other biopsychosocial variables that influence the severity and prognosis of the condition (Phillips et al., 2010; Sarrami et al., 2017; Merckelbach et al., 2019). In this regard, pain, in particular cervical pain, is one of the most common symptoms in the aforementioned condition and one of the most influential factors in its prognosis (Ferrari et al., 2005; Mankovsky-Arnold et al., 2014). Patients who suffer more intense and persistent pain may have a slower and worse recovery process (Casey et al., 2015), and the perception of long-lasting extensive pain is associated with greater disability, depression, and poorer self-efficacy (Holm et al., 2008; Carroll et al., 2009; Falla et al., 2016; Aguilera et al., 2019).

However, the relation of pain and other variables like the above-mentioned is not unidirectional. Psychological and social factors like anxiety, depression, illness perception, disability perception, health-related quality of life (HRQoL), or poorer self-efficacy are especially relevant in the clinical experience and presentation of pain (Wallin and Raak, 2008; Linton and Shaw, 2011; Skaer and Kwong, 2017; Campbell et al., 2018). For example, evidence indicates that the presence of high-severity pain may produce a traumatic condition that generates anxious-depressive symptomatology, and the presence of such symptomatology leads patients to adopt maladaptive coping styles that generate a negative emotional state in which more attention is paid to pain, which serves to exacerbate it (Aparicio et al., 2013; Lumley et al., 2011).

In order to make a complete diagnosis of all the factors influencing WAD, the evidence suggests that its medico-legal evaluation should follow an integrated biopsychosocial model (Phillips et al., 2010; Gopinath et al., 2015; Walton and Elliott, 2017). This perspective would offer a fuller view of WAD, recognizing the interplay between the biomechanical, medical, psychological, and social factors (Turk et al., 2018).

Despite the progress made on the subject, the evidence of which psychosocial factors influence the perception of pain in litigant WAD patients is limited. Knowing these factors would help to understand the different perception profiles in which the condition may manifest and to provide treatment and medico-legal assessment tailored to the particular needs of each case.

The main objective of the present study is to analyze the perception of pain in a forensic population of victims of MVA reporting WAD. The specific objectives are: (a) to derive psychosocial subgroups of MVA victims using an exploratory cluster analysis; (b) to investigate the differences of the obtained subgroups in several outcomes (pain, disability) of sociodemographic and medico-legal variables; and (c) to observe possible differences in the profiles of pain found in this medico-legal sample (litigant patients) and the clinical samples described in the literature.

## Materials and Methods

### Study Design and Participants

A cross-sectional (observational and analytical) design was used with participants recruited from a multidisciplinary medical center specializing in bodily injury assessment of the Region of Murcia (Spain), during the years 2017–2019. Patients went to the clinic to be assessed after suffering an MVA.

During the study period, participants underwent a full clinical evaluation by one of three physicians who were experts in bodily injury assessment and who participated in the research. At the end of this evaluation, the experts invited the patients who met the selection criteria (specified in the following section) of the study to participate, providing them with a detailed explanation of its purpose and procedure, and emphasizing its anonymous and voluntary nature. Those individuals who agreed to participate signed the informed consent and were assessed by a forensic psychologist who applied the prepared scale battery.

To perform this study, we followed the ethical considerations proposed by the American Psychological Association (2017) and the favorable report of the Coordinating Council of the Ph.D. programs of Murcia.

We used as case selection criteria: (a) victims of MVA (car crash); (b) adults (≥18 years); (c) submitting to a medico-legal assessment by one of the three medical experts who participate in this study, to ensure their experience in injury assessment (avoiding malingering cases) and data reliability; (d) diagnosed with WAD (Pastakia and Kumar, 2011; Represas et al., 2020); (e) established causality nexus between the MVA and injury consolidation, according to forensic standards (Magalhães and Vieira, 2014); (f) without another kind of acute cervical injury or chronic disease (namely degenerative pathology), or other medical condition, to avoid response distortion of the applied tools; and (h) submitting to a psychological assessment, always by the same forensic psychologist, to ensure data reliability. A total of 249 subjects were chosen for the study.

### Variables and Measures

For this study, the variables of interest were divided into two groups: (a) Clustering variables, used in the cluster analysis to form the subgroups, considering health-related quality of life, presence and severity of depression, presence and severity of anxiety, and cognitive and emotional representations of illness and (b) profiling variables, with which the subgroups resulting from the prior analysis were compared, which include pain perception, perception of disability, sociodemographic variables, and variables of medico-legal interest.

#### Clustering Variables

*Health-related quality of life (HRQoL)*, measured by the 36-Item Short Form Survey (SF-36; Ware, 2000). The SF-36 is a 36-item with the following subscales: *Physical Function* (ability to perform physical tasks), *Role Physical* (capacity to fulfill one’s physical role), *Bodily Pain*, *General Health, Vitality* (energy/fatigue), *Social Function* (ability to perform activities and social tasks), *Role Emotional* (role limitations due to emotional problems), and *Mental Health*. Each one of the subscales yields a score from 0 to 100, with higher scores indicating the patient’s better quality of life. The Spanish version was used, with a general Cronbach’s alpha of 0.85 and 0.75 for all dimensions except for Social functioning (Alonso et al., 1995). For our sample, the general Cronbach alpha was 0.90. All subscales were considered and assessed, except for Bodily Pain because it was redundant due to using the *Brief Pain Inventory*.

*Presence and severity of depression*, measured by the Beck Depression Inventory (BDI-II; Beck et al., 1996). The BDI-II is a 21-item, self-report inventory with a score ranging from 0 to 63. Total score of 0–13 is considered minimal range, 14–19 is mild, 20–28 is moderate, and 29–63 is severe. As it is a short scale with good psychometric properties (α = 0.86), it can be especially useful in the medico-legal context. In this study, we used the Spanish version of Sanz et al. (2003), with an alpha of 0.86. For our sample, the Cronbach alpha was α = 0.83. In the results and discussion, we will refer to this instrument as the BDI.

*Presence and severity of anxiety*, measured by the Beck Anxiety Inventory (BAI; Beck et al., 1988). The BAI is a 21-item, self-report inventory with a score ranging from 0 to 63. The scores are classified as minimal anxiety (0–7), mild anxiety (8–15), moderate anxiety (16–25), and severe anxiety (30–63). Like the BDI, it is a short scale with good psychometric properties (α = 0.94). We used the Spanish version of Sanz and Navarro (2003), with an alpha of 0.86. For our sample, the Cronbach alpha was α = 0.87. In the results and discussion, we will refer to this instrument as the BAI.

*Cognitive and emotional representations of illness*, measured by the Brief Illness Perception Questionnaire (Brief IPQ; Broadbent et al., 2006). The BIPQ, a brief version of the Illness, Perception Questionnaire (Weinman et al., 1996), is a 9-item, self-report inventory designed to assess the cognitive and emotional representations of illness. Each item evaluates these dimensions on a score ranging from 1 to 10. As authors state, “it allows very simple interpretation of scores: increases in item scores represent linear increases in the dimension measured (p. 635).” Internal consistency was not evaluated in the study used to design the scale, but it showed high convergent validity with the IPQ (Broadbent et al., 2006). The Emotional representation and Consequences subscales were not considered because they were redundant due to using the BDI, BAI, and SF-36. We used the Spanish version of Pacheco-Huergo et al. (2012), with an alpha of 0.67. For our sample, the Cronbach alpha was α = 0.82.

#### Profiling Variables

*Pain severity and maximum/minimum pain*, measured with the Brief Pain Inventory (BPI; Cleeland, 1989, 1990, 1991; Cleeland and Ryan, 1994). The BPI is an 11-item, self-report measure, where each item is rated from 0 (*no pain*) to 10 (*extreme pain*) on a visual analog scale (VAS) that was developed to allow the patients to measure the severity of the clinical pain suffered, as well as the degree of social disturbance (Cleeland, 1991). Cronbach alpha ranges from α = 0.77 to 0.91. We used the Spanish version of Llach et al. (2003), with alphas of 0.87 and 0.89. For our sample, the Cronbach alpha was α = 0.89.

*Perception of disability*, measured with the Neck Disability Index (NDI; Vernon and Mior, 1991). The NDI is a 10-item, self-report measure that was developed to allow patients to provide information on how the pain has affected the social dimension and their perception of disability, with a score ranging from 0 to 34. The scores are classified as 0–4 = no disability; 5–14 = mild; 15–24 = moderate; 25–34 = severe; above 34 = complete. Unlike the Interference dimension of the BPI, which seeks to evaluate the alteration caused by pain in general, the NDI is designed to specifically measure the interference in the different social areas caused by pain in the cervical region. It has high internal consistency with a Cronbach alpha of α = 0.92. For our sample, the Cronbach alpha was α = 0.93.

#### Other Variables of Interest

An *ad hoc* questionnaire was developed to collect the sociodemographic variables of the participants (*sex and age*) and medico-legal interest (t*ime of day when the accident occurred, seat occupied in the vehicle, location of impact, type of road, seat belt fastened, head position, state of the car after the collision, time from the accident until the medico-legal evaluation, and symptomatology described*). The inclusion of the variable “speed of the car at the moment of impact” was initially considered, but it was discarded due to the impossibility of determining it objectively. To determine the causal link, we used the four criteria set out in Section 135 of Spanish Law 35/2015 of the scale of traffic accidents: (a) exclusion, which means that there is no other cause that fully justifies the injury/symptomatology; (b) chronological, which consists of the symptomatology appearing within a medically explainable time; (c) topographic, which means that there is a relationship between the body area affected by the accident and the injury/symptomatology referred unless a pathogenic explanation justifies otherwise; and (d) intensity, consisting of the adequacy between the injury/symptomatology referred and the mechanism of the trauma, taking into account the intensity of the accident and the other variables affecting the likelihood of the symptom’s existence. To consider the existence of a causal nexus, all the above criteria must be met.

### Data Analysis

Statistical analyses were performed using SPSS software version 25. Descriptive and frequency statistics were obtained from the total sample, and significant differences in the clustering and sex profiling variables were studied using one-way analysis of variance (ANOVA).

Three steps were performed for the cluster analysis: (1) determination of the optimal number of clusters. For this purpose, the procedure of Aguerrevere et al. (2018) was followed by applying the two-step autoclustering analysis of the SPSS, which selects the optimal number of clusters by taking the highest ratio of distance measures (RDM) and the largest change information criterion measure on the Schwarz’s Bayesian Criterion (change BIC); (2) cluster analysis with the clustering variables. The K-means procedure was used with the clustering variables and the optimal number of clusters indicated by the previous analysis; and (3) cross-validation. For this purpose, the differences in the profiling variables were examined with an ANOVA with Tukey HSD *post hoc* tests for the continuous variables and the chi-squared test for the categorical variables. Discriminant analysis was then performed with a stepwise method on the variables used to form the clusters, thus evaluating the ability of that group of variables to predict group membership.

Due to differences in the format of the scale results, the clustering variables were standardized as Z-scores. After performing these analyses, the Z-scores were converted to the original values of each scale to facilitate the presentation and comprehension of the results.

## Results

### Analysis of the Sample

The assessment was carried out after an average of 38.24 days (SD = 9.25) since the accident, with a range of 26–60 days. Participants had a mean age of 36.36 years (SD = 10.81; range of 18–60), and 51% were men (*n* = 126). All claimed to suffer from cervical pain, with an average severity of 6.17 (SD = 0.92) in *BPI*, 96.1% (*n* = 237) excessive sensitivity of the cervical region, 20.6% (*n* = 51) dizziness, and only 3.3% (*n* = 8) of the sample expressed another symptom in addition to those mentioned, with 11 cases (4.4%) reporting pain in the lumbar area. None of them referred suffering from pain in any other part of the body, tinnitus, ocular pain, neurological symptoms, or gastrointestinal disorders. MVA-related data are described in Table 1.

**TABLE 1:** Collision-related factors in whiplash claimants.

Collision factors	*n*	%
**Moment of the day the collision occurred**		
Daytime	176	70.6
Nighttime	65	26.1
At dawn	5	2
At dusk	3	1.3
**Seat occupied**		
Driver’ seat	60	24.2
Front seat occupant	82	32.7
Back seat occupant	107	43.1
**Impact location**		
Front	55	22.2
Rear	173	69.3
Side	21	8.5
**Type of road**		
Rural	26	10.5
City	171	68.6
Highway	52	20.9
**Seat belt fastened**		
No	15	5.9
Yes	234	94.1
**Position of the head**		
Forward	200	80.4
Turned to the left	26	10.5
Turned to the right	23	9.2
**Car status after the accident**		
Irreparable damage	7	2.6
Serious damage	29	11.8
Average damage	177	71.2
Minor damage	36	14.4

### Cluster Analysis

#### Optimal Number of Clusters

A two-step analysis was applied with the autoclustering option on the entire sample. The results indicated that the 3-cluster solution was the most suitable (BIC = 916.42, RDM = 1.88) because the 2- and 4-cluster solutions obtained a higher BIC and a lower RDM (BIC = 932.63, RDM = 1.80 and BIC = 950.19, RDM = 1.78, respectively). The Silhouette measure of cohesion and separation considered the cluster quality as “fair” (0.87).

#### Comparison of Groups Based on the Clustering Variables

A K-means cluster analysis was performed for the variables shown in Table 2. The distribution of patients within the 3-cluster solution was: Cluster 1 had 72 participants (28.92%), Cluster 2 had 103 (41.37%), and Cluster 3 had 74 participants (29.72%).

**TABLE 2:** Means, SD, and ANOVAs of the cluster analysis.

	Cluster 1 (*N* = 72)	Cluster 2 (*N* = 103)	Cluster 3 (*N* = 74)	*F* (2, 246)	*p*	Significant intercluster differences (*p* < 0.05)
Depression (BDI)	1.92 (2.35)	4.78 (3.54)	22.17 (3.84)	816.96	*	3 > 2, 3 > 1, 2 > 1
Anxiety (BAI)	2.74 (3.88)	3.75 (2.89)	18.17 (3.41)	561.62	*	3 > 2, 3 > 1
Role emotional (SF-36)	78.77 (12.97)	58.68 (15.42)	37.22 (17.42)	132.92	*	3 > 2, 3 > 1, 2 > 1
Vitality (SF-36)	67.95 (16.89)	37.22 (15.24)	27.11 (15.02)	136.29	*	3 > 2, 3 > 1, 2 > 1
Mental health (SF-36)	96.35 (7.10)	84.20 (15.41)	53.92 (19.12)	160.24	*	3 > 2, 3 > 1, 2 > 1
Social function (SF-36)	69.98 (15.88)	51.12 (14.27)	33.87 (12.71)	116.01	*	3 > 2, 3 > 1, 2 > 1
General health (SF-36)	77.82 (12.71)	57.19 (15.35)	43.05 (14.95)	106.08	*	3 > 2, 3 > 1, 2 > 1
Physical function (SF-36)	53.13 (13.91)	39.94 (11.49)	26.46 (11.22)	87.74	*	3 > 2, 3 > 1, 2 > 1
Role physical (SF-36)	73.91 (12.42)	72.94 (12.82)	55.17 (18.40)	40.37	*	3 > 2, 3 > 1
Timeline (BIPQ)	3.85 (2.01)	4.32 (2.25)	6.57 (2.98)	27.10	*	3 > 2, 3 > 1
Personal control (BIPQ)	6.55 (1.42)	6.71(1.55)	6.15 (2.10)	2.38	0.09	n.s.
Treatment control (BIPQ)	6.22 (1.34)	6.47 (1.28)	6.12 (1.83)	1.32	0.26	n.s.
Identity (BIPQ)	2.31 (1.47)	2.54 (1.61)	2.45 (1.98)	0.39	0.67	n.s.
Coherence (BIPQ)	6.91 (1.55)	6.55 (1.77)	5.11 (2.37)	18.67	*	3 > 2, 3 > 1
Concern (BIPQ)	3.09 (2.32)	2.98 (1.81)	3.44 (2.35)	1.03	0.35	n.s.

BPI, Brief Pain Inventory; NDI, Neck Disability Index; BDI, Beck Depression Inventory; BAI, Beck Anxiety Inventory; SF-36, 36-Item Short Form Health Survey; BIPQ, Brief Illness Perception Questionnaire.

*p < 0.001; n.s., not significant.

Cluster 1 (C1) includes patients who obtained overall low scores in all the analyzed variables and present no anxious-depressive symptomatology (BAI, *M* = 2.74, SD = 3.88; BDI, *M* = 1.92, SD = 2.35) and low alteration of HRQoL (Role Emotional, *M* = 78.77, SD = 12.97; Vitality, *M* = 67.95, SD = 16.89; Mental Health, *M* = 96.35, SD = 7.10; General Health, *M* = 77.82, SD = 12.71; and Role Physical, *M* = 73.91, SD = 12.42), except for a medium alteration of Physical and Social Functioning (*M* = 53.13, SD = 13.91 and *M* = 69.98, SD = 15.88, respectively).

Cluster 2 (C2) is characterized by overall scores of low-medium severity, with medium-low alteration of HRQoL (Role Emotional, *M* = 58.68, SD = 15.42; Vitality, *M* = 37.72, SD = 15.24; Mental Health, *M* = 84.20, SD = 15.41; Social Function, *M* = 51.12, SD = 14.27; General Health, *M* = 57.19, SD = 15.35; and Physical Function, *M* = 39.94, SD = 11.49), significantly higher than the C1 group except for Role Physical (*M* = 72.94, SD = 12.82), and presenting no anxious-depressive symptomatology (BAI, *M* = 3.75, SD = 2.89; BDI, *M* = 4.78, SD = 3.54).

Cluster 3 (C3) is characterized by higher scores than the other two clusters in the studied variables, with the exception of the variables in which no differences were found. The results showed that this group presents anxious-depressive symptomatology (BAI, *M* = 18.17, SD = 3.41; BDI, *M* = 22.17, SD = 3.84), medium-high alteration of the HRQoL variables, measured by the SF-36 (Role Emotional, *M* = 37.22, SD = 17.42; Vitality, *M* = 27.11, SD = 15.02; Mental Health, *M* = 53.92, SD = 19.12; Social Function, *M* = 33.87, SD = 12.71; General Health, *M* = 43.05, SD = 14.95; Physical Function, *M* = 26.46, SD = 11.22; Role Physical, *M* = 55.17, SD = 18.40), worse knowledge about the condition (Coherence, IPQ, *M* = 5.11, SD = 2.37) and worse prognosis of improvement (Timeline, IPQ, *M* = 6.57, SD = 2.98).

Except for the variables indicated in C1 (timeline and coherence), the three groups have a similar profile in the illness perception variables of the BIPQ, with a medium-high control capacity, both for treatment and personal control, low concern about the disease, and good identity of symptoms.

#### Differences in Subgroups in the Profiling Variables

the analysis of the subgroups produced by cluster analysis based on the variables of medico-legal and sociodemographic interest is presented in Table 3. In terms of pain, significant differences were found depending on the severity of the pain, *F*(2, 246) = 103.43, *p* = 0.00; maximum pain, *F*(2, 246) = 65.75, *p* = 0.00; minimum pain, *F*(2, 246) = 16.38, *p* < 0.001; perception of disability, *F*(2, 246) = 35.73, *p* < 0.001; and time elapsed since the accident, *F*(2, 246) = 16.24, *p* = 0.00. No significant differences were found as a function of age, *F*(2, 246) = 0.29, *p* = 0.74.

**TABLE 3:** Means, SD, and ANOVAs of the profiling variables.

	Cluster 1 (C1)	Cluster 2 (C2)	Cluster 3 (C3)	*F* (2,246)	*p*	Significant Intercluster Differences (p < 0.05)
Pain severity (BPI)	3.97 (1.37)	4.63 (1.07)	6.70 (0.98)	103.43	*	3 > 2, 3 > 1, 2 > 1
Maximum pain (BPI)	6.27 (1.11)	7.04 (1.29)	8.71 (1.13)	65.75	*	3 > 2, 3 > 1, 2 > 1
Minimum pain (BPI)	2.14 (1.39)	2.62 (1.37)	3.66 (1.32)	16.38	*	3 > 2, 3 > 1, 2 > 1
Disability perception (NDI)	13.62 (5.00)	17.39 (4.67)	22.09 (3.82)	35.73	*	3 > 2, 3 > 1,2 > 1
Time since the injury	59.00 (22.01)	41.82 (23.89)	38.91 (23.79)	16.24	*	3 < 1, 2 < 1
Age	36.84 (9.79)	37.93 (12.21)	36.27 (11.50)	0.29	0.74	n.s.

BPI, Brief Pain Inventory; NDI, Neck Disability Index.

*p < 0.001; n.s., not significant.

The subgroups were also analyzed as a function of the variables related to the accident and sex, finding no significant differences in any of them (Time of day of the collision: χ^2^ = 6.25, *p* = 0.39, Wilks λ = 0.20; Seat occupied: χ^2^ = 8.27, *p* = 0.08, Wilks λ = 0.23; Location of the impact: χ^2^ = 0.77, *p* = 0.92, Wilks λ = 0.07; Road type: χ^2^ = 4.39, *p* = 0.17, Wilks λ = 0.35; Seat belt fastened: χ^2^ = 0.32, *p* = 0.84, Wilks λ = 0.04; Head position: χ^2^ = 5.52, *p* = 0.23, Wilks λ = 0.19; Car Status: χ^2^ = 4.40, *p* = 0.62, Wilks λ = 0.17; Sex: χ^2^ = 2.65, *p* = 0.26, Wilks λ = 0.13).

#### Discriminant Analysis (Cross-Validation)

To validate the cluster solution obtained with the methodology presented in the previous section, discriminant analysis was performed with the variables that the cluster analysis had used to form the subgroups: Depression (Wilks λ = 0.66, *p* < 0.001), Anxiety (Wilks λ = 0.83, *p* < 0.001), Role Emotional (Wilks λ = 0.47, *p* < 0.001), Vitality (Wilks λ = 0.55, *p* < 0.001), Mental Health (Wilks λ = 0.51, *p* < 0.001), Social Function (Wilks λ = 0.55, *p* < 0.001), General Health (Wilks λ = 0.75, *p* < 0.001), Physical Function (Wilks λ = 0.60, *p* < 0.001), Role Physical (Wilks λ = 0.55, *p* < 0.001), Timeline (Wilks λ = 0.42, *p* = 0.004), and Coherence (Wilks λ = 0.46, *p* = 0.006).

The analysis showed two functions with significant results for variables in Functions 1 to 2 (χ^2^ = 280.32, *p* = 0.00, Wilks λ = 0.14) and in the Function of test 2 (χ^2^ = 21.64, *p* < 0.001, Wilks λ = 0.86).

These results indicated that the variables used could explain the relationship between the variables and the subgroups created by the cluster: 96.8% (Functions 1 and 2, canonical correlation = 0.91) and 3.2% (Function 2, canonical correlation = 0.37). Stronger relationships for Function 1 were observed in Role Emotional, Mental Health, and BDI and for Function 2, in Role Physical and Social Function. Finally, cross-validation of the classification showed that the model could correctly classify 96.1% of the 3 subgroups (97.2% of Cluster 1, 97.3% of Cluster 2, and 93.2% of Cluster 3).

## Discussion

The main objective of the present study was to analyze the perception of pain in victims of MVA suffering from WAD. We evaluated the factors that have been cited most frequently as contributing to WAD symptoms, as well as WAD-related impairment and disability (Carroll et al., 2009). The study represents one of the first attempts to establish the contribution of biomedical and psychosocial factors to WAD-related pain in a medico-legal sample of patients who have been determined to be accurately reporting their WAD symptoms.

Concerning the specific objectives of this work, through the results of the cluster analysis, three different groups (C1, C2, and C3) of WAD patients were found using psychosocial variables as the clustering input. Group C3 is composed of patients who show a general moderate-high overall severity profile, consistent with the acute phase profile presented by Sterner and Gerdle (2004). Anxious-depressive symptomatology and moderate-high alteration of HQoL and of the cognitive representation of the disease are observed. This alteration of HQoL is manifested in a decrease in the ability to properly perform physical tasks and social activities, loss of vitality, and feeling fatigue, limits in the performance of the usual role due to physical and emotional problems, and perception of general health alteration. Similarly, these patients have developed a maladaptive cognitive representation of the disease, characterized by a slightly poorer comprehension of the condition suffered, as well as the belief that it would be extended over a long period of time. On the other hand, in the profiling variables, this group presents significantly higher values of overall pain severity, maximum and minimum pain, as well as a higher perception of disability.

Group C2 is composed of patients who have moderate general severity, with no anxious depressive symptomatology and a moderate-low alteration of HQoL/cognitive representation of the disease. Alterations in physical and social function, vitality, and Role Emotional are observed, but these patients are more capable of performing the activities that make up their Role Physical than the C3 group. Similarly, they show a more adaptive cognitive representation of the disease than the other two groups.

Pain severity and perception of intermediate disability, less than group C3 but significantly higher than the third and final group (C1) were observed. The C1 group is composed of patients with low scores in the presented variables, but who still have some alteration in HQoL, especially in Vitality and the Social and Physical Functions, and suffer mild pain and alteration in the perception of disability.

Significant differences were also observed depending on the time elapsed since the accident. In Group C1, which shows mild symptoms, the average time was significantly higher than in the other two. This suggests that the severity of symptoms decreases two or three months after their origin, but some symptoms, such as pain, persist. These results coincide with the synthesis of Carroll et al. (2009), where recovery times of between 1 and 6 months were recorded, with pain being the symptom that takes the longest to disappear.

On the other hand, we consider of interest the absence of significant differences between groups C2 and C3 in this variable. These two groups show a similar time since the accident just over 1 month—but they have a different severity profile. As no differences were observed in the collision variables, a possible explanation for this difference in profiles is the interaction between the psychological variables and pain. The presence of the psychological symptoms may provoke a higher perception of pain in the patient because of the attentional bias that they can cause (Arola et al., 2010; Ho et al., 2018). Depression and anxiety are important mediators in catastrophizing, hypervigilance, and avoidance—variables associated with an exacerbation of the perception of pain. Patients with anxious-depressive symptomatology tend to catastrophize as an adaptation strategy, producing a state of hypervigilance or a negative emotional state in which more attention is paid to pain, which worsens it (Aparicio et al., 2013; Lumley et al., 2011). The interaction may also occur in the opposite direction because the evidence of the direction of the relationship between pain and these variables in WAD patients is inconclusive (Woo, 2010). Thus, the severity of high pain can lead to an experience of the traumatic condition that generates psychological symptoms (Phillips et al., 2010). These results confirm the need for the evaluation of WAD cases to be as personalized and detailed as possible.

Group differences in the perception of disability can also be explained from the influence of psychological variables. The presence of anxious-depressive symptomatology is associated with the avoidance of activity and a perception of high disability (Zale and Ditre, 2015). Patients who suffer higher pain restrict their movements and activity to avoid the emergence of pain, which would lead to a greater perception of disability (Vlaeyen and Linton, 2000). Likewise, these restrictions on mobility can result in a decrease in physical abilities due to lack of use, which would increase the patient’s feeling of incapacity (Kasch et al., 2001). For this reason, we consider it very important for patients to receive psychoeducation that provides them with anxious-depressive-symptom management tools, as well as information about possible cognitive distortions related to the disease process. It would also be advisable to provide a training plan that includes exercises to strengthen and rehabilitate the affected areas (for example, State Insurance Regulatory Authority, 2014).

Concerning the other variables studied in the comparison of the subgroups, no significant differences were found in the factors related to the accident (time of day, seat occupied, impact location, road type, fastened seat belt, head position, and car condition), indicating that these variables do not influence the severity of symptoms. These findings are consistent with the results obtained by other authors, where these variables have not been observed to be associated with the prognosis of the condition (Sterling and Kenardy, 2011).

On the other hand, no significant differences were found depending on age or sex in any of the variables studied. These results coincide with the recent study of Ickmans et al. (2017), where no difference was found based on personal factors, and that of Malfliet et al. (2015), where no differences were found as a function of sex in psychological variables such as depression, fear, somatization, physical function, physical pain, or general health. As these authors point out, while evidence shows that women have a higher incidence of WAD, these results indicate that their psychosocial profile of the condition is not different from that of men (Malfliet et al., 2015).

Generally speaking, our results show a WAD pain profile consistent with what was previously presented in the bibliography (Sullivan et al., 2002; Åhman and Stålnacke, 2008; Beltran-Alacreu et al., 2018). It is a condition with general pain of intermediate-high degree, which can reach very high values in specific situations, such as making a sudden movement or excessive effort. Despite this, patients can control the severity of pain to some extent, mainly through the use of medication, as can be seen in the recorded minimum pain scores of the BPI and the Treatment Control of the BIPQ. On the other hand, the presence of psychological symptoms is also consistent with the results of Carroll et al. (2009), which showed that a high percentage of WAD patients developed anxious-depressive symptomatology at 6 weeks and manifested a worse quality of life. These results are also consistent with the experience of pain in a musculoskeletal injury (Ottosson et al., 2007) and coincide with the reports of other authors in the study of cervical pain and HRQoL, in which it is stated that pain in the cervical area decreases the range of movements that the person can perform and may produce functional limitations in the correct performance of daily activities, which may translate into an alteration of the patients’ quality of life (Leaver et al., 2013; Pedisic et al., 2013).

We consider that the three groups obtained may be of interest to the medico-legal context, as they provide evidence of the different litigant WAD patient profiles that the professional may encounter. As can be seen, the classification performed by the cluster analysis indicates that some patients have a pattern of symptoms that could be considered atypical, which is not explained by the time elapsed since the accident or the influence of factors related to the collision. As noted above, this magnification of symptoms is often interpreted as a sign of malingering and can create a false alarm effect in which the litigant patient is considered to be exaggerating the symptoms to obtain greater financial compensation, with the psychological and social consequences that this entails (Thompson et al., 2018). As Merckelbach et al. (2019) explain, not only the patient’s type of symptoms should be considered, but also when and how they occur, so the professional should properly analyze and evaluate all the possible hypotheses. In this sense, we explained previously that malingering or cyclic interaction between pain and psychosocial symptomatology are two possible explanations for symptoms of unusual severity, but other factors such as the item sequence and the tests applied, confusion or lack of information about symptoms or the disease process, inattentive response style or personality traits can also be considered (Merckelbach et al., 2019).

Similarly, legislation for the management of road accidents in the country to which the person being assessed belongs is also an issue that needs to be analyzed. Represas et al. (2008) found that there was much more incidence of WAD in Galicia, an autonomous community in northeastern Spain, than in the north-central part of Portugal, even though the population characteristics were very similar. They considered as a possible explanation the fact that “the Spanish legislation allows the direct conversion of easily simulated symptoms to a system of legal points, resulting in much greater economic compensation than in Portugal (p. 355).”

We believe that the evaluation of the litigant WAD patient should be conducted from a multidisciplinary and biopsychosocial approach (e.g., Albert et al., 2003). This perspective offers a fuller view of it, recognizing the interplay between the medical, biomechanical, social, and psychological factors (Turk et al., 2018). In this way, the professional will be able to offer appropriate action guidelines to the specific characteristics of each case and with sufficient evidence of the reason for possible overreporting of symptoms.

## Limitations

The results obtained in this study should be interpreted according to several limitations. Due to the conditions and design used, it was not possible to use probabilistic sampling. Another limitation of the study is that we could not follow up the patients evaluated to check the interaction between pain severity and the selected variables. We consider that studying this interaction could be useful to identify possible modulating variables. Also, the cut-off points used belongs to a spanish sample and this should be taken into account when considering the implications of the results. Finally, due to limitations imposed by the medical center, we had a short lapse of time to apply the evaluation protocol, so it was not possible to include other variables of interest like pain catastrophizing.

## Conclusion

The cluster analysis shows three different profiles and a symptomatic heterogeneity, which supports the evaluation of WAD from the biopsychosocial model of disease and the evaluation of pain from the multidimensional model. The comparison of the groups showed that the patients of the group of high-perceived pain presented a profile of greater severity in the psychosocial variables studied. Given these results, we consider that the biopsychosocial evaluation of WAD, with a multidisciplinary approach, could be useful to obtain a complete view of it, avoiding diagnostic errors between genuine patients and malingerers, and ensuring that the treatment follows an approach adapted to the real needs of the patient.

## Data Availability Statement

The raw data supporting the conclusions of this article will be made available by the authors, without undue reservation.

## Ethics Statement

The studies involving human participants were reviewed and approved by Consejo de coordinación del Programa de Doctorado de Ciencias Forenses de la Universidad de Murcia. The patients/participants provided their written informed consent to participate in this study.

## Author Contributions

DP, EP-L, and JR-H contributed to conception and design of the study. EP-L organized the database, performed the statistical analysis, and wrote the first draft of the manuscript. DP, AR-C, TM, and LA wrote sections of the manuscript. All authors contributed to manuscript revision, read, and approved the submitted version.

## Conflict of Interest

The authors declare that the research was conducted in the absence of any commercial or financial relationships that could be construed as a potential conflict of interest.

## Publisher’s Note

All claims expressed in this article are solely those of the authors and do not necessarily represent those of their affiliated organizations, or those of the publisher, the editors and the reviewers. Any product that may be evaluated in this article, or claim that may be made by its manufacturer, is not guaranteed or endorsed by the publisher.
